# Atmospheric CO_2_ Concentration and Other Limiting Factors in the Growth of C_3_ and C_4_ Plants

**DOI:** 10.3390/plants8040092

**Published:** 2019-04-04

**Authors:** Albert Boretti, Singarayer Florentine

**Affiliations:** 1 Department for Management of Science and Technology Development, Ton Duc Thang University, Ho Chi Minh City 700000, Vietnam; 2 Faculty of Applied Sciences, Ton Duc Thang University, Ho Chi Minh City 700000, Vietnam; 3 School of Health and Life Sciences, Federation University Australia, Ballarat, Victoria 3350, Australia; s.florentine@federation.edu.au

**Keywords:** CO_2_ enrichment, photosynthesis, carbon cycle, C_3_ species, C_4_ species

## Abstract

It has been widely observed that recent increases in atmospheric CO_2_ concentrations have had, so far, a positive effect on the growth of plants. This is not surprising since CO_2_ is an important nutrient for plant matter, being directly involved in photosynthesis. However, it is also known that the conditions which have accompanied this increase in atmospheric CO_2_ concentration have also had significant effects on other environmental factors. It is possible that these other effects may emerge as limiting factors which could act to prevent plant growth. This may involve complex interactions between prevailing sunlight and water conditions, variable temperatures, the availability of essential nutrients and the type of synthetic pathway for the plant species. The issue of concern to this investigation is if we should be worried about a possible shift in the C_3_-C_4_ paradigm driven by changes in the atmospheric CO_2_ concentration, or if some other factor, such as water scarcity, is much more relevant within a 30-year time frame. If an opinion is needed on what will have the worst effect on the survival of the planet between the scarcity of water or the reduced efficiency of C_3_ plants to sequester CO_2_, the issue of water is the more incisive.

## 1. Introduction

A higher concentration of carbon dioxide (CO_2_) in our atmosphere aids photosynthesis, thus contributing to increased plant growth. CO_2_ is essential for photosynthesis. By increasing the level of CO_2_, photosynthesis increases, but only if there is no other limiting factor. For example, nitrogen is often in short supply to control the amount of biomass produced. While it is certainly not appropriate to look at the CO_2_ fertilization effect in isolation, it is nonsense to claim that rising CO_2_ levels by itself might not be good for plants. While the indirect effects of an increasing CO_2_ concentration in the atmosphere may be not all positive, the direct effect of supplying one key nutrient within reasonable limits is undoubtedly always positive. 

**Figure 1:**
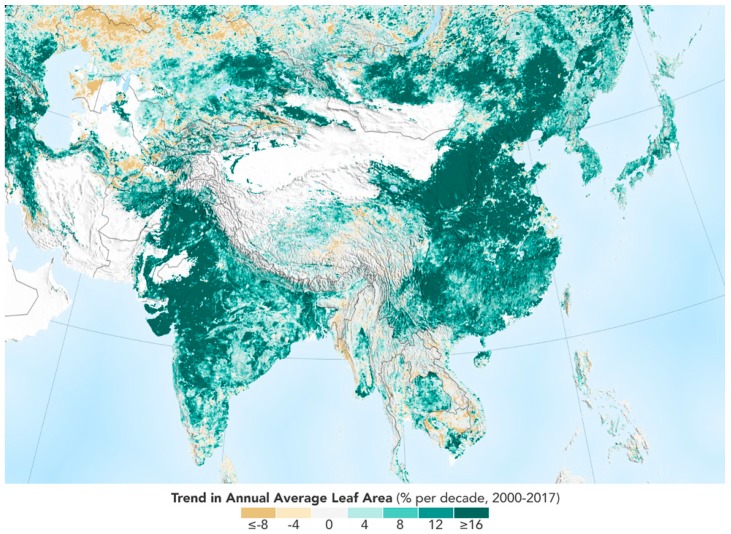
Trend in annual average leaf area from satellite. Image reproduced and modified after www.nasa.gov/sites/default/files/styles/full_width/public/thumbnails/image/asia_tamo_2017_full.png?itok=pSrXnDKA.

The recent finding by NASA [1] and [2] compared satellite data from the mid-1990s to today using high-resolution imagery. There has been a significant increase in greening around the world. It is unclear if the explanation is a warming planet, the increased CO_2_ concentration in the atmosphere, a wetter climate, or possibly better, a combination of the three, that has caused more plants to grow. As shown in Figure 1, the trend in annual average leaf area from satellite is mostly of growth. 

Reference [3] has recently proposed the result of an experiment that should support a reversal from the expected C_3_-C_4_ contrast, where theory and experiments have indicated, so far, that C_4_ species are less responsive to elevated carbon dioxide (eCO_2_) than C_3_ species. This work has been the subject of intense debate. Reference [4] challenged this result. Reference [5] partially agreed with the comment. However, in [6,7] the authors move further discussing these results, to claim that the rising CO_2_ levels might not be as good for plants as we thought, questioning the fact that the rising CO_2_ concentrations in the atmosphere produce faster growth of plants. References [8,9,10] further questioned the work [3]. The data collected for the paper [3], are deeply discussed here to demonstrate that there is no real indication that in the future the carbon cycle may not benefit from the availability of atmospheric CO_2_. Other issues, such as water scarcity, are more worrying than the alleged reduced efficiency of C_3_ plants to sequester CO_2_.

## 2. C_3_ and C_4_ Plants’ Biochemistry 

C_3_, C_4_ and Crassulacean acid metabolism (CAM) are the three different processes that plants use to fix carbon during photosynthesis. Approximately 95% of plant species on earth use the C_3_ photosynthetic pathway (C_3_ species), ~5% of plant species use the C_4_ photosynthetic pathway (C_4_ species), and a negligible percentage of plant species use the CAM photosynthetic pathway (CAM species). 

C_3_ and C_4_ plants differ for their photosynthesis. Green plants use sunlight to synthetize carbohydrates from atmospheric CO_2_, water and sunlight through photosynthesis. The general equation of photosynthesis

6·CO_2_ + 12·H_2_O → 6·C_6_H_12_O_6_ + 6·O_2_ + 6·H_2_O, (1)

holds for both C_3_ and C_4_ plants.

Photosynthesis is made of light dependent reactions and light independent dark reactions. In the light reactions, chlorophyll molecules absorb sunlight energy to synthesize chemical molecules and reduced coenzymes. In the dark reactions, these molecules then synthesize carbohydrates with CO_2_. 

C_3_ plants uses the C_3_ cycle of dark reaction to form a three-carbon compound as first stable product. C_4_ plants uses the additional dark reaction pathway C_4_ cycle to form a four-carbon compound as first stable product. 

While C_3_ plants have only one CO_2_ acceptor, C_4_ plants have two CO_2_ acceptors. While C_3_ plants perform photosynthesis only with stomata open, C_4_ plants perform photosynthesis also with stomata closed. While C_3_ plants have high photorespiration, photorespiration is absent in C_4_ plants. Under normal environmental conditions, the photosynthetic efficiency of C_3_ plants is consequently generally less than C_4_ plants. 

While the photosynthetic process is mostly driven by the energy, CO_2_ and H_2_O inputs, there are many environmental factors affecting the efficiency. For example, temperature, humidity or fertilizers. 

While C_3_ plants prefer cool (optimum temperature range of 18–24 °C) and wet areas, C_4_ plants prefer hot (optimum temperature range of 32–55 °C) and dry areas. Fertilizers such as nitrogen (N) or phosphorus (P) also have different effects on C_3_ and C_4_ plants.

## 3. General Factors Affecting Plant Growth 

It is discussed in general which are the factors affecting plant growth. So far, too much ambient CO_2_ has not been a direct reason for reduced plant growth. Conversely, the lack of water has always been a determinant factor preventing not only plant growth, but also plant life. 

There are in general four primary factors affecting plant growth, sunlight, water, temperature and nutrients, including CO_2_, nitrogen, phosphorus and others [11]. Three principal characteristics of sunlight that affect plant growth are quantity, quality and duration [12]. Temperature influences most plant processes, including photosynthesis, transpiration, respiration, germination and flowering [12]. Water and humidity also play a significant role, as most parts of the growing plants may be 90% in water. Water plays many roles in plants [12]. Water pH is also relevant [13]. Finally, plant nutrition is relevant. Plant nutrition is the basic chemical elements plants need. Fertilization refers to these materials added to the environment around a plant. The essential plant nutrients include carbon, oxygen and hydrogen, absorbed from the air, and other nutrients including nitrogen typically obtained from the soil. The most important mineral nutrients needed from their growing medium include macronutrients such as nitrogen (N), phosphorus (P), potassium (K), calcium (Ca), sulfur (S), magnesium (Mg), carbon (C), oxygen (O), hydrogen (H) and micronutrients such as iron (Fe), boron (B), chlorine (Cl), manganese (Mn), zinc (Zn), copper (Cu), molybdenum (Mo) and nickel (Ni) [14]. 

Even if hydrogen, oxygen, nitrogen and carbon contribute to over 95% of a plants’ biomass on a dry matter weight basis, a nutrient deficiency can limit growth and yield. Thus, the soil fertility must be modified through the addition of a fertilizer to promote growth and increase or sustain yields [15]. 

There is an ever-expanding archive of the results of peer-reviewed scientific studies that report on the growth responses of plants to atmospheric CO_2_ enrichment, in terms of either dry weight (biomass) or photosynthesis (net CO_2_ exchange rate) [16]. Plant photosynthesis and dry weight responses to atmospheric CO_2_ enrichment are proven to be positive for almost every plant in the world [16]. Satellite monitoring [17], has shown significant greening over the last 35 years from a quarter to half of Earth’s vegetated lands that is largely due to rising levels of atmospheric CO_2_. 

## 4. A Possible Reversal of C_3_-C_4_ Grasses Responses to Elevated CO_2_

Reference [3] recently proposed the result of an experiment that should support a reversal from the expected C_3_-C_4_ contrast. 

Over the first 12 years of their specific 20-year free-air CO_2_ enrichment experiment with 88 C_3_ or C_4_ grassland plots, in [3] it was stated that biomass was noticeably enhanced at eCO_2_ relative to ambient CO_2_ in C_3_ but not C_4_ plots, while during the subsequent eight years, they stated that biomass was noticeably enhanced at eCO_2_ relative to ambient CO_2_ in C_4_ but not C_3_ plots. According to the authors, these findings challenge the current C_3_-C_4_ eCO_2_ paradigm and show that even the best-supported short-term drivers of plant response to global change might not predict long-term results. 

The work of [3] has been used to cast doubts about the direct, positive effect that an increasing atmospheric CO_2_ level may have on the growth of plants, namely favoring one species take-over of the other.

It would have been interesting, to understand the way the authors in [3] decided and controlled all the parameters affecting the biomass production during their 20-year long experiment. This may have permitted to understand the reproducibility, and therefore the reference value, of their results, voiding the otherwise very likely opportunity of unaccounted or undisclosed biases being responsible for their unexpected trend. 

Apart from the unaccounted or undisclosed other parameters affecting the C_3_ and C_4_ plants growth in the specific experiment, what it is shown in the paper is not even what is concluded in the paper, and even less what is suggested by the comments by [7] or [6].

**Figure 2:**
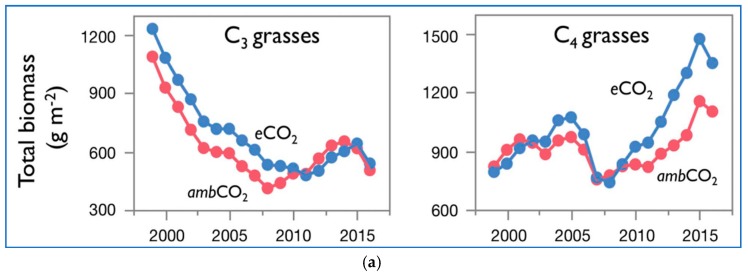

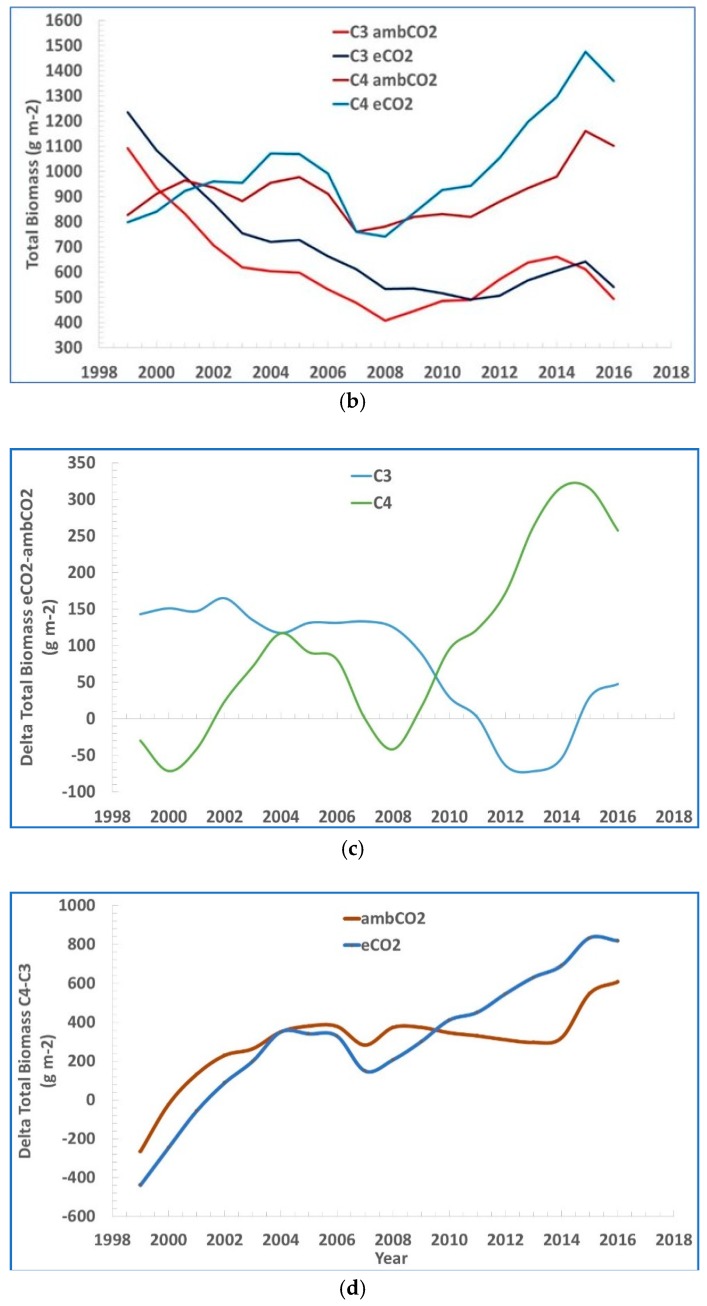
Biomass of C_3_ grasses and C_3_ grasses at ambient and elevated (+180 ppm) CO_2_ from 1998 to 2017. (**a**) Total biomass (aboveground +0 to 20 cm belowground) of plots comprising C_3_ grasses and C_4_ grasses in ambient CO_2_ (red) and elevated CO_2_ (blue) vs. time. Each point represents data pooled across N treatments, and across monoculture and four-species plots (equally weighted), for each functional group (*n* = 22 plots for each functional group at each CO_2_ level). Image reproduced modified from [3] Reich, P.B., Hobbie, S.E., Lee, T.D. and Pastore, M.A., 2018, Unexpected reversal of C3 vs.C4 grass response to elevated CO_2_ during a 20-year field experiment, 360(6386): 317–320, doi:10.1126/science.aas9313. Reprinted with permission from AAAS. (**b**–**d**) The same data digitized and further analyzed here. Results are proposed as total biomasses in the same graph, delta total biomass eCO2 – ambient CO_2_ for C_3_ and C_4_ plants, and eCO_2_, and delta total biomass C_4_-C_3_ for ambient CO_2_ and eCO_2_.

Figure 2a presents the biomass over time of C_3_ grasses and C_4_ grasses of [3] at ambient and elevated CO_2_. The data are shown as moving three-year averages centered over the middle of each three-year group. In Figure 2b–d their data of total biomass of both C_3_ and C_4_ plants vs. time is coupled together and further analyzed. 

The C_3_ grasses’ graphs show a declining trend in the total biomass. The decline is continuous, 1997 to 2011 in the case of eCO_2_, and 1997 to 2008 in the case of ambCO_2_, from values of about 1250 and 1100 g/m^2^, down to values of about 500 and 400 g/m^2^. Then, the total biomass starts a reverse trend, up to about 2015, going back to about 650 g/m^2^ for both eCO_2_ and ambCO_2_. The differences between eCO_2_ and ambCO_2_ are small. The very last point shows a further decline for both eCO_2_ and ambCO_2_ to about 500 g/m^2^. 

The C_4_ grasses’ graphs show a rising trend in the total biomass. From about 800 g/m^2^ for both eCO_2_ and ambCO_2_, the total biomass increases first up to a relative maximum around 2005 of 1100 and 900 g/m^2^ respectively for eCO_2_ and ambCO_2._ Apart from the peak region, the curves overlap. Then there is a generalized downturn up to values of about 700 g/m^2^ around 2007 for both eCO_2_ and ambCO_2_, and then a rising trend stronger for eCO_2_ than ambCO_2_, up to values of 1500 and 1150 g/m^2^ around 2015. The last point is characterized by a now reducing total biomass for both eCO_2_ and ambCO_2_. 

The difference in total biomass eCO_2_ – ambCO_2_ is generally positive in both C_3_ and C_4_. It is decreasing over time in C_3_ and it is increasing in C_4_, but it is negative only over the time window 2011–2014 in C_3_, and over the two time-windows 1999–2001 and 2007–2009 in C_4_.

A larger total biomass measured with eCO_2_ vs. ambCO_2_, is mostly decreasing with time in case of C_3_ grasses, and mostly increasing in time in the case of C_4_ grasses, with a possible explanation being, apart from the experiment uncertainties, the additional parameters affecting the process not accounted or not disclosed in the study.

The difference between the total biomass C_4_ – C_3_ increases steadily in time for both eCO_2_ vs. ambCO_2_.

## 5. A Brief Literature Review of the Possible Reversal of the C_3_-C_4_ Plants Paradigm

Reference [4] challenged this result arguing the responses of C_3_ and C_4_ plants to elevated CO_2_ observed in [3] can be explained by the natural history of the plants and soils in the experiment without challenging the C_3_-C_4_ elevated CO_2_ paradigm. 

Reference [5] partially agreed with the comment. They did not agree with the assertion that such explanation make their results irrelevant for questioning the C_3_-C_4_ elevated CO_2_ paradigm based on the C_3_-C_4_ differences in the photosynthetic pathway.

Reference [3] attracted other comments, such as in [8,9,10] reported here after.

Reference [8] noticed about [3], that it would have been interesting to discuss their control of all the other parameters affecting the biomass production during their 20-year long experiment. This would have made it possible to understand the reproducibility of their results, voiding the likely opportunity of unaccounted biases not mentioned in the paper. 

Reference [8] also commented the inappropriateness of questioning the fact that the rising CO_2_ concentrations in the atmosphere results in faster growth of plants as done by [7]. Results from a single, unclear study, were indeed used by [7] to cast doubts about future crop production and changes of ecosystems following the increasing levels of atmospheric CO_2_.

Reference [9] noticed about [3] that their unexpected findings about the long-term response of C_3_ and C_4_ species under eCO_2_, were statistically not very convincing and a comprehensive explanation of the observed phenomena was missing. The claim that C_4_ species became more responsive to eCO_2_ than C_3_ species, and produced more biomass after long-term exposure, was associated with increases in soil nitrogen mineralization after long exposure; however, results were obtained under no or relatively low level of nitrogen supply [9].

Reference [9] also noticed that [3] did not support soil N mineralization with data on depletion of nitrogen and other resources in the soil and resource uptake by the plants. Additionally, in [3] they did not relate leaf nitrogen content to photosynthesis. Reference [9] concluded that unfortunately, the work only showed a small part of the big picture, making impossible to produce any better assessment.

Reference [10] also expressed their concern about the study [3]. Reference [10] highlighted the uncertainties associated with the long-term study. Reference [10] also highlighted the “over-interpretation” of the results.

Reference [10] noticed that while the biomass of both C_3_ and C_4_ species changed substantially over time, the changes in soil N mineralization over time did not agree with the trends in biomass. 

Reference [10] observed as the first down trend, then up trend, near the end, for the biomass for the C_3_ species, as well as the more complicated pattern for the C_4_ species, were not explained.

The plant nitrogen uptake could have explained the results. However, there were no measurements of the plant nitrogen uptake. If the level of soil nitrogen and trace minerals is not measured over time, then it is not possible to understand what the limiting factor is. 

Reference [10] noted the data on leaf-level photosynthesis did not indicate a switch between C_3_ and C_4_ groups in responding to eCO_2_.

Reference [10] remarked that the results of the two nitrogen supply treatments presented separately could have been relevant. If the nitrogen availability was behind the response, the response to eCO_2_ should have been different between the nitrogen treatments. 

Reference [10] also mentioned that [3] did not give any information on the differential changes to soil nitrogen mineralization resulted from the changed soil microbial community. Reference [3] only suggested a gradual decrease in critical soil nutrients throughout the most part of the 20-year long experiment for the C_3_ group, and a different behavior for the C_4_ group.

Finally, the authors of [10] suggested the secondary effects of eCO_2_ on transpiration and canopy temperature as a further, possible explanation of the results. 

It is known, as in [18] or in [19], that relative to C_3_ species, C_4_ species respond more to eCO_2_ in decreasing stomatal conductance and transpiration. This difference can result in changes in soil moisture dynamics and soil temperature profile, and thus likely in soil microbial community and activity and soil nitrogen mineralization in the long term [20]. Again, no data on soil moisture and temperature is presented by [3].

According to [10], the results shown in [3] did not represent causality. More background data with the required explanatory parameters measured or presented could have permitted to resolve the unusual C_3_ and C_4_ response over time. 

Reference [10] does not believe the data presented in [3] supports the claim that their results challenge the current C_3_-C_4_ eCO_2_ paradigm. This paradigm is defined by the primary physiology of C_3_ and C_4_ photosynthesis, namely the absence in C_3_ vs. the presence in C_4_ leaves of the CO_2_-concentrating mechanism that suppresses photorespiration, as shown in [21] or in [22]. 

Reference [10] noted that secondary effects are often associated with plant response to eCO_2_ over a longer term, as shown in [23]. The secondary response of the leaf area index to eCO_2_ may overcome the primary photosynthetic response to eCO_2_, as shown in [24] or in [25]. This only stresses the need of improved models for simulating the leaf area index in response to eCO_2_. In the opinion of [10], if the trends reported in [3] are general, there is no need to hypothesize a paradigm change, but to improve the modelling of the biogeochemical ecosystem to include secondary effects.

## 6. Future Environmental Constraints: Water Scarcity as the Most Likely Limiting Factor

While there is no direct negative effect of ambient CO_2_ enrichment on plant growth, this does not mean that other environmental issues will not limit plant growth soon. One major environmental issue that is being strongly downplayed is water scarcity. 

It is the claim of [26] that water scarcity will be a significant issue in the next few decades, more significant on a regional, than a global scale. Reference [26] also reports that water scarcity will be made worse by global warming’s effects on drought and rainfall on a regional scale. With an increasing population, and an increasing GDP, both driving the demand for agriculture products and water, and the increment of water and air pollution, it is thus very likely that, regionally, domestic consumption will compete with agricultural consumption.

Last year has been the year of the largest ever use of fresh water, and very likely, the year with the largest ever pollution of fresh water [26]. Water scarcity is already an issue, in many areas of the world, that will become more and more significant in the next few decades, with excessive population and economic growth two of the drivers, as global fresh water use, and pollution, rises as a function of population growth, economic development and changing consumption patterns. With scarcity at a localized level likely much harder than scarcity at regional or country level, there are areas, especially of Africa, where the most part of the population growth is expected, where droughts will get worse. Very likely, most places will run out of water before they will run out of food, and there is a clear risk of eating seeds in time of famine, i.e., people will need to drink the water and not pour it on crops, with significant consequences on agriculture production. 

The latest concentration measured by the Mauna Loa Observatory, Hawaii (www.co2.earth) is 409.23 ppm. This is a local, not a global measure [27,28,29], and a likely overrating of the global average CO_2_ concentration, as Mauna Loa is one active shield volcano of five volcanoes that form the Island of Hawaii, historically considered the largest volcano on Earth. CO_2_ concentrations have a time and space variability [27,28,29], that is often neglected.

**Figure 3:**
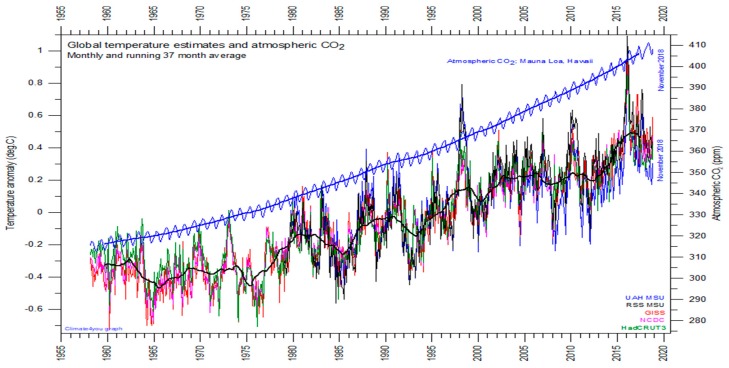
Global temperature records, quality class 1 satellite temperature records such as University of Alabama at Huntsville (UAH), USA, quality class 2 reconstructed temperature records such as HadCRUT, a cooperative effort between the Hadley Centre for Climate Prediction and Research and the University of East Anglia’s Climatic Research Unit (CRU), UK, and quality class 3 reconstructed temperature records such as National Climatic Data Center (NCDC), USA and Goddard Institute for Space Studies (GISS), at Columbia University, New York City, USA and the atmospheric CO_2_ measured at the Mauna Loa Observatory. Image reproduced modified after www.climate4you.com/images/ AllCompared%20GlobalMonthlyTempSince1958%20AndCO2.gif.

According to the latest estimations of the Intergovernmental Panel on Climate Change (IPCC) Fifth Assessment Report (www.ipcc.ch/assessment-report/ar5/), the long-term change in global temperature that would result from doubling the atmospheric CO_2_ concentration between 1.5 and 4.5 °C. Figure 3 is a graph of global temperature records, quality class 1 satellite records such as University of Alabama at Huntsville (UAH), USA, quality class 2 such as HadCRUT, a cooperative effort between the Hadley Centre for Climate Prediction and Research and the University of East Anglia’s Climatic Research Unit (CRU), UK, and quality class 3 records such as National Climatic Data Center (NCDC), USA and Goddard Institute for Space Studies (GISS), at Columbia University, New York City, USA, and the atmospheric CO_2_ measured at the Mauna Loa Observatory. 

For the three reconstructed surface air temperature records, HadCRUT but especially NCDC and GISS, administrative changes of the values are quite often introduced, even for observations many years back in time. These reconstructions are based on a blend of sea surface data collected by different means, plus data from land stations that have sometimes moved geographically and had their instrumentation changed, of unknown quality, and degree of representativeness. These uncertainties determine the quality classes of www.climate4you.com.

Over this 60-year time-window, the global temperatures have warmed of 0.6 °C, while the CO_2_ concentration has increased by 90 ppm. 

Within another 60-year time-window, we may thus reasonably expect a warming of less than 0.6 °C, and a further increase of CO_2_ concentration of less than 90 ppm. Not this temperature warming, nor this increment of CO_2_ concentration, is expected to affect agriculture production to a major extent. Conversely, water scarcity may be much harder than thought within this time window, if the population and gross domestic product (GDP) continues to grow at the same rate, and similarly water uses and pollution. 

**Figure 4:**
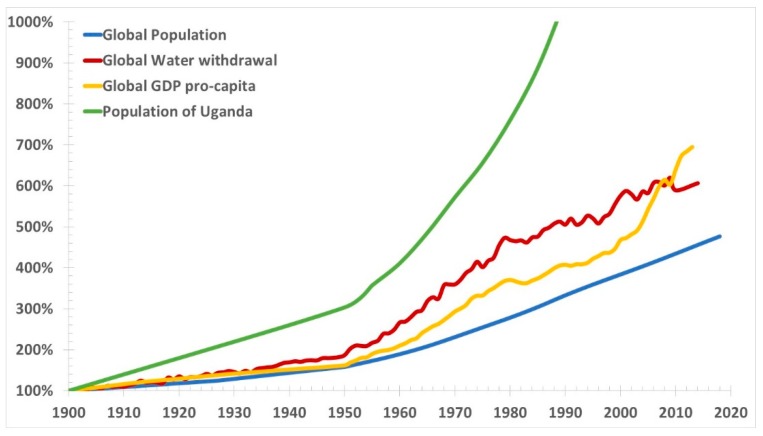
Global gross domestic product (GDP) pro-capita, water withdrawals and population, and the population of Uganda from 1900 to present. As global debt pro-capita is only available since 1950, it is not shown in the graph. However, as it has growth steadily to the latest 225% of the GDP, it would be mostly off-scale. Local, more than global, patterns are of concerns for water scarcity, where exponentially growing demand of fresh, clean water must be satisfied by a similarly exponentially reducing availability of fresh, clean water.

Figure 4 presents global GDP pro-capita, water withdrawals and population, and population of Uganda from 1900 to present. 

Water withdrawal is an incorrect measure of water scarcity, as the increasing water consumption is accompanied by the similarly increasing pollution and reduction of fresh water reserves that is not accounted in the graph. Aquifers are shrinking. They are also subjected in coastal areas to salt water intrusion, because of the excessive water withdrawal induced subsidence coupled to thermosteric sea level rise. Pollution is also generally growing. 

Population growth inevitably leads to greater water consumption, and reduced water availability. While the rich near the coast can use desalination, albeit at significant energy costs, there are masses of poor people remote from the sea—especially in Africa which is the fastest growing population—where desalination does not help. They have already had many droughts and they will get worse. It is impossible to legislate for rain, and groundwater is rapidly depleted, and polluted. Very likely, most places will run out of water. 

Regarding the GDP, it must be added that the International Monetary Fund (IMF)’s Global Debt Database [30] indicates that the debt globally in 2017 has reached an all-time high of $184 trillion, or 225% of GDP. The world’s debt now exceeds $86,000 per capita, which is more than 250% of the average income per-capita. The most indebted economies in the world are the richer ones. The United States, China, and Japan account for more than half of global debt, exceeding their share of global output. 

It is therefore questionable to claim that the current economic model is beneficial to the economy, while it is certainly wrong to claim that this model is beneficial to the environment, as the excessive growth of the population and GDP is producing an anything but sustainable consumption, shrinking and pollution of the fresh water resources. Water scarcity driven by the population and economic growth may affect agriculture, which is supposed to use a larger share of land to cope with the increasing food demand, much earlier than the time when the anthropogenic CO_2_ emissions will produce significant warmings, well before 2050 in many areas of the world.

## 7. Conclusions

There is no proof in the data presented in [3] that in the future most of the Earth’s plants might not soak up as much CO_2_ as previously expected, while some grasslands might take up more. There is a present trend where land plants have scrubbed about one third of the CO_2_ that humans have emitted into the air producing more biomass. However, the results proposed in [3] may not be simply dismissed as either a set-up specifically designed to produce the contrary results, or results that are not reproducible. If the C_3_ plants will be less efficient than the C_4_ plants in the use of CO_2_, as has been suggested by [3], this is a phenomenon to be considered. Reference [3] deserves the credit for having opened a window on a relevant issue, namely that certain effects cannot be measured with short-term experiments, but require multi-year, costly and difficult to manage experiments.

Many acknowledge that global warming caused by CO_2_ emissions will contribute to increasing water stress, that is also driven by the poor management of the fresh water resources and the growth of the population and economy. If an opinion is needed on what will be the worst effect on the survival of the planet between the scarcity of water or the possibly reduced efficiency of C_3_ plants to sequester CO_2_, obviously, the issue of water is the more incisive. There is certainly the need of a more equilibrate global picture to cope with the many environmental threats we do have, by considering the possible effect of water scarcity on food production as a priority.
